# Cost-effectiveness of cerebrospinal biomarkers for the diagnosis of Alzheimer’s disease

**DOI:** 10.1186/s13195-017-0243-0

**Published:** 2017-03-16

**Authors:** Spencer A. W. Lee, Luciano A. Sposato, Vladimir Hachinski, Lauren E. Cipriano

**Affiliations:** 1 Ivey Business School, Western University, 1255 Western Road, London, ON N6G 0N1 Canada; 2 School of Medicine, University College Cork, College Road, Cork, T12 YN60 Ireland; 3 Department of Clinical Neurological Sciences, London Health Sciences Centre, Western University, London, ON N6A 5A5 Canada; 4 Stroke, Dementia & Heart Disease Laboratory, Western University, London, ON N6A 5A5 Canada; 5 Department of Anatomy & Cell Biology, Western University, London, ON N6A 5A5 Canada; 6 Department of Biostatistics and Epidemiology, Schulich School of Medicine and Dentistry, Western University, London, ON N6A 5C1 Canada

**Keywords:** Alzheimer’s disease, Cost-effectiveness analysis, Cerebrospinal fluid biomarkers, Neuroimaging

## Abstract

**Background:**

Accurate and timely diagnosis of Alzheimer’s disease (AD) is important for prompt initiation of treatment in patients with AD and to avoid inappropriate treatment of patients with false-positive diagnoses.

**Methods:**

Using a Markov model, we estimated the lifetime costs and quality-adjusted life-years (QALYs) of cerebrospinal fluid biomarker analysis in a cohort of patients referred to a neurologist or memory clinic with suspected AD who remained without a definitive diagnosis of AD or another condition after neuroimaging. Parametric values were estimated from previous health economic models and the medical literature. Extensive deterministic and probabilistic sensitivity analyses were performed to evaluate the robustness of the results.

**Results:**

At a 12.7% pretest probability of AD, biomarker analysis after normal neuroimaging findings has an incremental cost-effectiveness ratio (ICER) of $11,032 per QALY gained. Results were sensitive to the pretest prevalence of AD, and the ICER increased to over $50,000 per QALY when the prevalence of AD fell below 9%. Results were also sensitive to patient age (biomarkers are less cost-effective in older cohorts), treatment uptake and adherence, biomarker test characteristics, and the degree to which patients with suspected AD who do not have AD benefit from AD treatment when they are falsely diagnosed.

**Conclusions:**

The cost-effectiveness of biomarker analysis depends critically on the prevalence of AD in the tested population. In general practice, where the prevalence of AD after clinical assessment and normal neuroimaging findings may be low, biomarker analysis is unlikely to be cost-effective at a willingness-to-pay threshold of $50,000 per QALY gained. However, when at least 1 in 11 patients has AD after normal neuroimaging findings, biomarker analysis is likely cost-effective. Specifically, for patients referred to memory clinics with memory impairment who do not present neuroimaging evidence of medial temporal lobe atrophy, pretest prevalence of AD may exceed 15%. Biomarker analysis is a potentially cost-saving diagnostic method and should be considered for adoption in high-prevalence centers.

**Electronic supplementary material:**

The online version of this article (doi:10.1186/s13195-017-0243-0) contains supplementary material, which is available to authorized users.

## Background

Alzheimer’s disease (AD) is a progressive neurodegenerative disorder currently affecting an estimated 36 million people globally, with prevalence predicted to double in the next 10 years [1–3]. In the United States alone, with 5.2 million patients with AD [4], total direct costs in 2014 were estimated to be $214 billion, with another $220 billion in unpaid care [1]. Accurate and timely diagnosis of AD is important to initiate treatment promptly and to avoid inappropriate therapeutic interventions in patients with false-positive diagnoses [5]. Even though current treatments (acetylcholinesterase inhibitors and memantine) do not reverse the underlying neurological damage, AD treatments can delay cognitive and functional decline and improve overall quality of life [6, 7]. Several studies have found AD treatments to be cost-effective in mild to moderate AD and moderate to severe AD [8–11].

Clinical diagnosis of AD has a relatively low and highly uncertain diagnostic accuracy [12, 13]. To aid in diagnosis, neuroimaging by computed tomography (CT) or magnetic resonance imaging (MRI) is typically performed, both to rule out non-AD causes of cognitive impairment, such as meningioma and subdural hematoma, and to evaluate structural indicators of AD, including medial temporal lobe (MTL) atrophy [14]. Still, these neuroimaging techniques do not provide the desired level of accuracy to confidently diagnose AD in a considerable proportion of patients. Single-photon emission computed tomography (SPECT), ^18^F-fluorodeoxyglucose positron emission tomography (PET), and amyloid PET are effective at ruling out a diagnosis of neurodegenerative disease and amyloid-β (Aβ) deposition in the brain, but the results are complex, difficult to interpret, and have low to moderate positive predictive value, especially in older patients because brain Aβ deposition increases with age [14–16].

Cerebrospinal fluid (CSF) biomarkers have demonstrated relatively high diagnostic accuracy even for prodromal AD in patients with mild cognitive impairment (MCI) [14, 15] and so may provide additional diagnostic insight. However, CSF collection involves a lumbar puncture, which has an associated cost and causes patient discomfort.

Previous cost-effectiveness analyses of AD diagnostic technologies present conflicting findings potentially attributable to differences in the clinical setting of the diagnosis being considered [17, 18]. In two studies performed in the early 2000s, researchers found the addition of SPECT and PET to clinical assessment was not cost-effective [19, 20]. Authors of a recent cost-effectiveness analysis compared clinical assessment plus florbetapir-PET with clinical assessment alone and found the addition of florbetapir-PET to be cost-effective from the perspective of the Spanish National Health System [21]. However, they did not compare PET with a standard diagnostic regimen including CT or MRI analysis. Researchers in a cost minimization study, also performed from the perspective of the Spanish National Health System, suggested that the use of CSF biomarkers may reduce AD-related health care costs [22]. However, that study did not account for the discomfort and risks of undergoing lumbar puncture or improvements in quality of life for patients accurately diagnosed with AD. In the present study, we evaluated the cost-effectiveness of performing CSF biomarker analysis in a cohort of patients with suspected dementia who were referred to a neurologist or memory clinic and who remained without a definitive diagnosis after neuroimaging.

## Methods

**Fig. 1:**
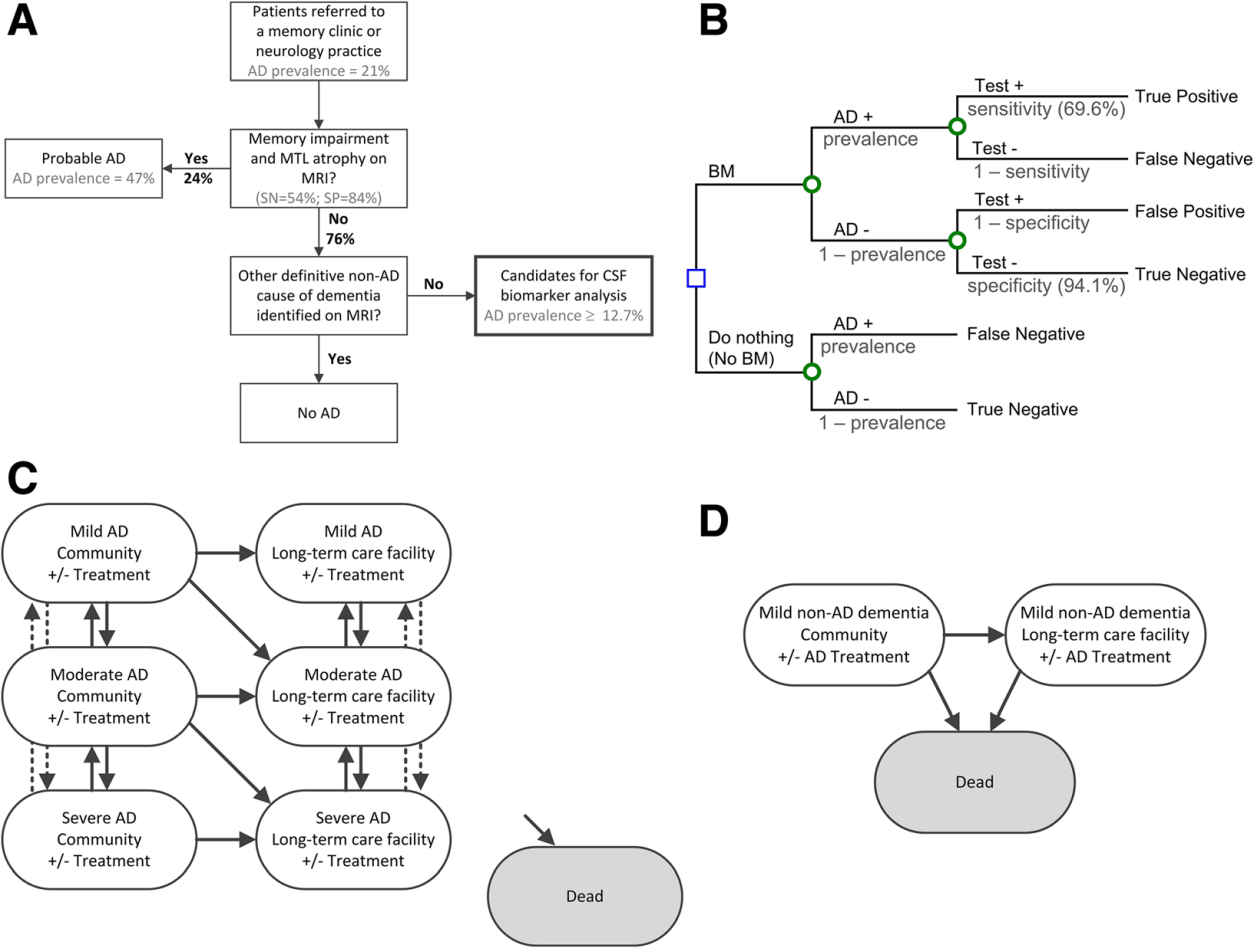
Patient cohort and decision model. **a** Flowchart of patients referred to a memory clinic or neurology practice with suspected dementia, some of whom would be diagnosed with probable AD after clinical assessment and MRI. If MRI does not provide a definitive non-AD diagnosis for any patients, and if all patients remain candidates for biomarker analysis, the pretest prevalence of AD is 12.7%. If MRI provides a definitive non-AD diagnosis for some patients, the pretest prevalence of AD in the cohort patients who continue to have suspected AD is greater than 12.7%. **b** Schematic of a decision tree. The *blue square* represents the decision whether to use CSF biomarkers. *Green circles* represent chance events. The population is divided into four groups on the basis of whether the patients have Alzheimer’s disease and the outcome of the diagnostic strategy: true-positive, false-negative, false-positive, and true-negative. The proportion of patients in each group is determined by the prevalence of AD in the evaluated cohort and the sensitivity and specificity of each diagnostic strategy. **c** Markov model of Alzheimer’s disease. Patients begin in the health states for community-dwelling mild, moderate, and severe AD. Each month, patients may die, progress or regress in terms of disease severity, discontinue or reinitiate treatment, transition from living in the community to living in a long-term care facility, or stay in the same health state. In the model, patients living in a long-term care facility cannot return to living in the community. **d** Markov model for patients with non-AD dementia. Patients begin in the community-dwelling non-AD dementia state. Only those with a false-positive diagnosis of AD receive AD treatment. Each month, patients may die, discontinue or reinitiate treatment, transition from living in the community to living in a long-term care facility, or stay in the same health state. *AD* Alzheimer’s disease, *BM* Biomarker, *CSF* Cerebrospinal fluid, *MRI* Magnetic resonance imaging, *MTL* Medial temporal lobe, *SN* Sensitivity, *SP* Specificity

We developed a Markov model to evaluate the lifetime costs and benefits of performing CSF biomarker analysis in patients referred to a neurologist or memory clinic with suspected dementia who, after evaluation by neuroimaging, do not have a definitive diagnosis of AD or another cause of dementia (Fig. 1a). In 1-month time steps, the model followed the diagnosis and health state progression of a hypothetical cohort of patients. We used standard health economic methods by taking a societal perspective, considering costs and benefits over a lifetime horizon, discounting costs and benefits at 3% annually, and performing both probabilistic and deterministic sensitivity analysis to evaluate the robustness of our findings [23]. For determining cost-effectiveness, we used the commonly applied thresholds of $50,000 and $100,000 per quality-adjusted life-year (QALY) gained [24]. We implemented the model in Microsoft Excel 2013 using Visual Basic for Applications (Microsoft Corp., Seattle, WA, USA).

### Model overview

A schematic of the model is presented in Fig. 1. We considered two diagnostic strategies: biomarker analysis and do nothing. Patients were divided into four groups on the basis of their true health state and diagnosed health state: true-positive, false-negative, false-positive, and true-negative (Fig. 1b). Similar to previously published model-based analyses of AD [19], individuals who had AD were divided into 12 health states on the basis of the severity of their disease, whether or not they were on treatment, and their location (Fig. 1c). In the base case analysis, we assumed that patients who did not have AD had another disease causing stable MCI, so individuals who did not have AD were divided into four health states on the basis of whether they were on AD treatment (because of false diagnosis) and their type of residence (Fig. 1d). We performed structural sensitivity analysis exploring alternative assumptions for the natural history for the non-AD patients, including modeling it as a stable, moderate cognitive impairment and as a progressive cognitive impairment with transition rates similar to AD.

In each month, patients could die or transition from one health state to another. We estimated the rate of transition between disease states, the influence of treatment on those transitions, as well as costs and utilities associated with each health state from the medical literature (Table 1). When multiple sources were available to inform parameters, we selected studies that were more generalizable to the modeled population (i.e., large, U.S.-based cohorts) and those using more recent datasets. When the literature reported conflicting evidence or wide uncertainty, we selected a central value for the base case and performed extensive sensitivity analysis over the entire range of values reported in the literature. We validated model outcomes by replicating the analysis of previously published model-based cost-effectiveness studies of AD diagnosis [20, 25].

**Table 1:** Base case inputs, ranges for sensitivity analysis, and sources

Parameter	Base case	Low value	High value	Source [reference]
Patient population				
Start age, years	65	55	75	[4]
Initial AD severity distribution (%)				
Mild	70	0.5400	0.783	[67]
Moderate	28	0.1850	0.427	[67]
Severe	2	0.0170	0.033	[67]
Diagnosis				
Diagnostic test accuracy				
Status quo: clinical assessment plus MR neuroimaging (CA + MR)				
Sensitivity (SN_MR_)	0.54	0.46	0.62	[13]
Specificity (SP_MR_)	0.84	0.79	0.89	[13]
Revised criteria: clinical assessment plus MR neuroimaging and/or biomarker analysis				
Sensitivity (SN_MR+BM_)	0.86	0.80	0.92	[13]
Specificity (SP_MR+BM_)	0.79	0.74	0.84	[13]
Diagnostic accuracy of CSF biomarkers in patients with no medial temporal lobe atrophy on MRI				
Sensitivity (SN_BM|MR−_)	0.698	0.54	0.86	Calculated^a^
Specificity (SP_BM|MR−_)	0.941	0.89	0.98	Calculated^a^
Biomarker analysis				(see Methods)
Cost	463	250	600	[50]
QALY toll	−0.008	0	−0.02	[19, 57]
AD natural history model				
Mortality				
Age-specific mortality due to causes other than AD	Annual mortality rate = 3.53e^0.0909×Age^			Estimated^b^ [53, 68]
HRs for AD-specific mortality				
Mild	2.92	2.34	3.52	[29]
Moderate	3.85	2.94	5.05	[29]
Severe	9.52	6.60	13.4	[29]
Disease progression without AD treatment (annual rate per 100,000)				
From mild				
To moderate	27,710	24,939	30,481	[25, 31]
To severe	1385	1247	1524	[25, 31]
From moderate				
To mild	4478	4030	4925	[25, 31]
To severe	31,829	28,647	35,012	[25, 31]
From severe				
To mild	385	347	424	[25, 31]
To moderate	5332	4799	5865	[25, 31]
Transition to long-term care facility (annual rate per 100,000)				
From mild	2110	500	4000	[31, 43, 44]
From moderate	6957	1500	8000	[31, 43, 44]
From severe	11,747	2500	15,000	[31, 43, 44]
AD treatment				
Treatment uptake and adherence				
Treatment initiation				
Donepezil, at diagnosis	0.45	0.27	0.56	[37, 39, 41]
Memantine, at transition to severe AD	0.36	0.22	0.45	[38]
Treatment discontinuation (annual rate per 100,000)				
Donepezil, community dwelling	28,768	10,536	35,667	[36]
Donepezil, long-term care facility dwelling	62,362	51,083	69,315	[42]
Memantine	30,111	12,783	44,629	[6]
Treatment reinitiation after quitting (annual rate per 100,000)				
Donepezil	33,142	23,105	40,132	[38]
Memantine	22,314	17,834	25,541	[6]
Treatment effectiveness				
Donepezil HRs				
Transition from mild to moderate	0.5	0.253	0.989	[25]
Transition from moderate to mild	2.36	0.802	6.95	[25]
Transition from community to long-term care facility	0.37	0.2	0.5	[43]
Memantine				
Incremental utility (annualized)	0.051	0	0.1	[7]
HR, transition from community to long-term care facility	0.37	0.2	0.5	Assumed same as donepezil
Costs (US$)				
Age-specific baseline costs	Annual costs = 893e^0.0404×Age^			Estimated (see Methods)
45–64 years	5499	4000	8000	[51]
65–84 years	12,336	11,000	16,000	[51]
>84 years	27,674	25,000	34,000	[51]
Annual incremental costs by disease severity (including costs of informal caregiving)				
Community dwelling				
Patients without AD	24,128	17,369	30,369	Assumed the same as Mild AD
Mild AD	24,128	17,369	30,369	(see Additional file 1)
Moderate AD	33,845	25,000	40,000	(see Additional file 1)
Severe AD	60,160	50,000	69,000	(see Additional file 1)
Long-term care facility dwelling				
Facility cost	83,950	70,000	95,000	[52]
Patients without AD	9872	7000	12,000	Assumed the same as Mild AD
Mild AD	9872	7000	12,000	(see Additional file 1)
Moderate AD	9872	7000	12,000	(see Additional file 1)
Severe AD	9847	7000	12,000	(see Additional file 1)
Medication (annual)				
Donepezil, 10 mg/day	2473	2000	4288	[69]
Memantine, 10 mg/day	3192	2500	5957	[69]
Age-specific annual health care costs in the year of death				
<90 years	35,158	32,000	39,500	[70]
>90 years	25,455	22,000	28,000	[70]
Utilities				
Age-specific weights				[54, 55]
60–64 years	0.83	0.822	0.835	
65–69 years	0.82	0.820	0.826	
70–74 years	0.81	0.803	0.818	
75–79 years	0.79	0.786	0.794	
>79 years	0.74	0.730	0.742	
Health state-specific weights				
Community dwelling				
Patients without AD	0.68	0.52	0.80	Assumed same as mild AD
Mild AD	0.68	0.52	0.80	[25]
Moderate AD	0.54	0.30	0.70	[25]
Severe AD	0.37	0.25	0.50	[25]
Long-term care facility dwelling				
Patients without AD	0.71	0.55	0.80	Assumed same as mild AD
Mild AD	0.71	0.55	0.80	[25]
Moderate AD	0.48	0.30	0.60	[25]
Severe AD	0.31	0.20	0.45	[25]

*Abbreviations: AD* Alzheimer’s disease, *BM* Biomarker, *CA* Clinical assessment, *CSF* Cerebrospinal fluid, *MR* Magnetic resonance, *MRI* Magnetic resonance imaging, *QALY* Quality-adjusted life-year, *SN* Sensitivity, *SP* Specificity

^a^The sensitivity of biomarker analysis in patients without abnormal medial temporal lobe atrophy on MRI (SN_BM|MR-_) was calculated using the sensitivity of the revised criteria (in which patients are diagnosed with AD if they have abnormal findings on MRI or abnormal biomarkers, denoted SN_MR+BM_) and the sensitivity of clinical assessment and MRI alone (SN_MR_) using the formula: SN_MR+BM_ = SN_MR_ + (1 − SN_MR_) × SN_BM|MR−_.The specificity of biomarker analysis in patients without abnormal medial temporal lobe atrophy on MRI (SP_BM|MR−_) was calculated using the specificity of the revised criteria (SP_MR+BM_) and the specificity of clinical assessment and MRI alone (SP_MR_) using the formula: SP_MR+BM_ = SP_MR_ × SP_BM|MR−_

^b^To avoid double-counting the deaths caused by AD, the age-specific mortality rate due to AD was subtracted from the all-cause mortality rate using an excess mortality model. The resulting “other-cause” age-specific mortality rate was smoothed using an exponential fit

### Data and assumptions

#### Patient cohort

The prevalence of AD in a cohort of patients with possible dementia varies across referral centers and increases with patient age and family history [1]. Of the 8495 patients referred to 30 U.S. Alzheimer’s disease centers, 24% were diagnosed with mild AD [26]. We estimated the true prevalence to be 21%, adjusting for the accuracy of diagnosis with clinical assessment and MRI (as the status quo), where proportion diagnosed = prevalence × sensitivity + (1 − prevalence) × (1 − specificity). Clinical assessment and MTL atrophy seen on MRI would help identify approximately half of the patients with AD in the referral population (sensitivity of memory impairment plus MRI is 54% [13]). Accounting for the diagnosis of AD after MRI, the approximate prevalence of AD in the remaining patients is 12.7% (Fig. 1a). In addition, MRI may provide another definitive diagnosis where the possibility of concomitant AD is highly unlikely and thus further consideration of AD using biomarkers is no longer clinically relevant. This patient selection will increase the pretest prevalence of AD (by which we mean the probability of AD in the cohort of patients with memory impairment, no abnormal MTL atrophy, and no alternative diagnosis precluding AD) in patients still considered candidates for biomarker analysis. Specifically, if 10%, 20%, or 40% of non-AD patients are correctly identified as having an alternative diagnosis (and not having AD) after MRI, then the pretest prevalence of AD increases to 14%, 15%, or 19%, respectively. Furthermore, if patients without memory impairment are excluded, the prevalence of AD in the cohort of patients considered for biomarker analysis increases to 39% (the sensitivity and specificity of memory impairment alone are 93% and 68%, respectively [13]). Variation in the case mix across referral centers, including the prevalence of AD and the distribution of causes for non-AD dementia, creates high uncertainty in the prevalence of AD in patients who remain without a definitive diagnosis after neuroimaging. Therefore, base case results are presented over the full range of possible AD prevalence.

#### Diagnostic accuracy

Bouwman et al. retrospectively evaluated the diagnostic accuracy of clinical assessment plus neuroimaging by MR and the revised AD diagnostic criteria [27] in 138 patients with AD and 223 memory clinic patients without AD [13]. Under the revised AD diagnostic criteria, patients were defined as having AD when clinical assessment indicated episodic memory impairment and either evidence of MTL atrophy and/or an abnormal biomarker profile [13, 27]. MTL atrophy was scored visually on a scale of 0 (no atrophy) to 4 (severe atrophy) for both left and right hippocampi and then averaged to generate a single score. Positive AD findings were based on age-specific thresholds: ≥1 was considered abnormal for patients aged <65 years; ≥1.5 was considered abnormal for patients aged 65–75 years; and >2 was considered abnormal for patients >75 years of age. For CSF biomarker analysis, CSF was obtained using a standard lumbar puncture procedure and measured by commercially available sandwich enzyme-linked immunosorbent assays. Positive AD findings based on CSF biomarkers required at least two of the three biomarker criteria to be satisfied: low Aβ_42_ concentrations (<495 ng/L), increased total tau concentrations (>356 ng/L), or increased phospho-tau concentrations (>54 ng/L). (For further information, refer to Bouwman et al. [13].)

We calculated the sensitivity and specificity of biomarker analysis performed in patients without evidence of MTL atrophy on MRI by solving for the values that would achieve the overall sensitivity and specificity observed using the revised AD diagnostic criteria. In sensitivity analyses, we considered a wide range of values for biomarker sensitivity and specificity after a normal MRI, with sensitivity ranging from 54% to 86% (base case 69.6%) and specificity ranging from 89% to 98% (base case 94.1%).

#### Mortality

All-cause mortality was estimated using 2009 U.S. life tables [28]. To estimate the total mortality rate for a patient with AD at each stage of the disease, we multiplied the age-specific mortality rate for death due to other causes by AD severity-specific mortality HRs [29]. In our model, AD treatments did not influence mortality, because an analysis of the National Alzheimer’s Coordinating Center (NACC) Uniform Data Set indicated that AD treatment did not influence the rate of death after adjusting for disease severity and other factors influencing treatment use [30].

#### Natural history of AD

Transition rates between AD severity health states and between living in the community to living in a long-term care facility (LTCF) were estimated using the NACC Uniform Data Set [31]. Despite the progressive nature of AD, this analysis and a similar analysis of the Consortium to Establish a Registry for Alzheimer’s Disease dataset estimated a positive probability of transitioning backward (e.g., from moderate to mild AD) [32]. Possible explanations for backward transition include variation in clinical presentation and assessment, concomitant disease, and treatment adjustments resulting in noisy observations over time or masking the true disease severity [32]. We used the severity-specific proportion of patients with AD on acetylcholinesterase inhibitor treatment and the HRs for progression on treatment to calculate treatment-stratified transition rates (details in Additional file 1: Section 1.1).

#### Treatment regimens, adherence, and efficacy

Treatment dosage and schedule were incorporated in accordance with various guidelines: donepezil 10 mg per day in mild and moderate AD [33–35] and memantine 10 mg per day in severe AD [33]. We represented all acetylcholinesterase inhibitors with donepezil because it is the most commonly prescribed of these drugs [36].

Acetylcholinesterase inhibitor uptake rates vary significantly across study cohorts, with initiation rates ranging from 27% [37] to 97% [38] in newly diagnosed patients with AD in the community. We estimated a moderate uptake rate of 45% on the basis of a study of community-dwelling patients who screened positive for dementia in a primary care setting [39]. Specialized or coordinated care increases treatment uptake rates [40]; therefore, we considered uptake rates from 27% to 56% in sensitivity analysis [37, 41]. Base case treatment discontinuation and reinitiation rates were informed by large observational cohorts such that 25% of community-dwelling patients and 46.4% of facility-dwelling patients discontinued AD treatment each year [36, 42], and 63% of community-dwelling patients and 36% of facility-dwelling patients who had discontinued AD treatments restarted treatment within 1 year [6, 38].

Consistent with previous model-based analyses of AD, acetylcholinesterase inhibitor treatment reduced the transition rate from mild AD to moderate AD and increased the transition rate from moderate AD to mild AD [25]. The benefit of memantine treatment was incorporated into our model by an improved quality of life for patients with severe AD by 0.051 QALYs per year, which we estimated on the basis of average improvement in activities of daily living reported in a meta-analysis [7]. In the base case, consistent with previously published model-based analyses of AD treatment [25], we assumed that donepezil treatment does not reduce the rate of transition between moderate and severe disease, although we explored this possibility in sensitivity analysis. In the model, patients not on AD treatment are 2.7 times more likely to transition to an LTCF, as specifically reported by authors of a large U.S. medical claims database analysis including more than 5000 patients with AD [43] and consistent with other literature reports [31, 44–46].

In the base case analysis, patients without AD received no benefits from AD treatment, but we varied this assumption in sensitivity analysis. Occupational or psychosocial treatments were not included in the model, because they likely incur similar costs and provide benefits to patients with AD dementia and non-AD dementia [47–49].

#### Costs

We identified the clinical visit and laboratory testing codes with the Healthcare Common Procedure Coding System (HCPCS) and Current Procedural Terminology (CPT), then we estimated their cost using the 2013 Medicare reimbursement schedule [50]. We assumed biomarker analysis required a lumbar puncture procedure for the collection of CSF (CPT code 62270), an immunoassay analysis (HCPCS code 83520), and a follow-up visit with a neurologist in which the diagnosis is reported (CPT code 99213), resulting in a total cost of $463.

In each month, individuals accrued age-specific health care costs unrelated to AD, additional AD severity-specific health care costs, and location-specific (community or LTCF) supportive care costs (paid and unpaid). Age-specific health care costs unrelated to AD, including out-of-pocket health care expenses, were based on the U.S. national average, which we smoothed using an exponential fit with a cap at the average annual cost of $33,870 for patients aged 90 years and older [51]. AD severity-specific costs of inpatient care, outpatient care, emergency care, and unpaid caregiving are detailed in Additional file 1: Section 1.2. The annual cost of living in an LTCF was estimated to be $83,950 (in 2013 U.S. dollars), based on the U.S. national average cost of a semiprivate room in a nursing home [52]. Costs were adjusted for inflation to constant 2013 U.S. dollars using the gross domestic product deflator [53].

#### Quality of life

We estimated baseline age-specific utilities from the Medical Expenditure Panel Survey data [54, 55]. Age- and AD severity-specific utilities were incorporated into the model by multiplying the age-specific utility by the AD severity-specific utility. Utility weights for each AD disease state were estimated on the basis of a prior cost-effectiveness analysis [25]. To our knowledge, no study to date has evaluated the one-time utility toll associated with embarrassment and discomfort before, during, and after a diagnostic test requiring lumbar puncture, including the risk and consequences of lumbar puncture-associated moderate to severe headache [56]. We assumed the one-time reduction in quality of life associated with lumbar puncture is approximately the same as the reduction in quality of life associated with breast biopsy, which has been measured to be equivalent to 2.92 quality-adjusted life-days (annualized to a one-time toll of 0.008 QALY incurred at the time of the test) [57].

### Analysis

We calculated the average lifetime discounted costs and QALYs for each diagnostic outcome and for each diagnostic strategy. If neither strategy cost less and provided more QALYs than the other, we calculated the incremental cost-effectiveness ratio (ICER). In a probabilistic analysis, we ran 10,000 independent simulations in which inputs were selected randomly from the probability distributions described in Additional file 1: Section 1.3 to determine 95% CIs for each outcome. We also performed deterministic sensitivity analyses to evaluate the robustness of our findings to uncertainty in model parameters and assumptions.

To provide general insight into the test characteristics that would make a new test or test combination both clinically and economically attractive after MRI, we identified the “challenge region” as described by Phelps and Mushlin at the willingness-to-pay (WTP) thresholds of $50,000 and $100,000 per QALY gained [58]. The boundary of the challenge region is identified as any set of new test characteristics, sensitivity $$ {\mathit{\mathsf{r}}}_1 $$ and specificity $$ {\mathit{\mathsf{r}}}_2 $$, for which the incremental net monetary benefit (INMB) compared with the current technology, with sensitivity $$ {\mathit{\mathsf{q}}}_1 $$ and specificity $$ {\mathit{\mathsf{q}}}_2 $$, at the WTP threshold (denoted λ) is greater than 0. The INMB comparing the two tests is calculated as

$$ \begin{array}{l}\mathit{\mathsf{INMB}}=\mathit{\mathsf{p}}\left({\mathit{\mathsf{r}}}_{\mathsf{1}}-{\mathit{\mathsf{q}}}_{\mathsf{1}}\right)\left[\lambda \left({\mathsf{QALY}}_{\mathsf{TruePositive}}-{\mathsf{QALY}}_{\mathsf{FalseNegative}}\right)-\left({\mathsf{Cost}}_{\mathsf{TruePositive}}-{\mathsf{Cost}}_{\mathsf{FalseNegative}}\right)\right]\\ {}\kern2.5em +\left(\mathsf{1}-\mathit{\mathsf{p}}\right)\left({\mathit{\mathsf{r}}}_{\mathsf{2}}-{\mathit{\mathsf{q}}}_{\mathsf{2}}\right)\left[\lambda \left({\mathsf{QALY}}_{\mathsf{TrueNegative}}-{\mathsf{QALY}}_{\mathsf{FalsePositive}}\right)-\left({\mathsf{Cost}}_{\mathsf{TrueNegative}}-{\mathsf{Cost}}_{\mathsf{FalsePositive}}\right)\right]\\ {}\kern2.5em -\Delta \mathsf{TestCost}-\lambda \Delta \mathsf{TestQALY}\end{array} $$

where $\mathit{\mathsf{p}}$ is the prevalence of the disease, ${\mathit{\mathsf{r}}}_1-{\mathit{\mathsf{q}}}_1$ is the improvement (or reduction) in sensitivity, ${\mathit{\mathsf{r}}}_2-{\mathit{\mathsf{q}}}_2$ is the improvement (or reduction) in specificity, $\left[\lambda \left({\mathsf{QALY}}_{\mathsf{TruePositive}}-{\mathsf{QALY}}_{\mathsf{FalseNegative}}\right)-\left({\mathsf{Cost}}_{\mathsf{TruePositive}}-{\mathsf{Cost}}_{\mathsf{FalseNegative}}\right)\right]$ is the INMB of preventing a false-negative diagnosis, $\left[\lambda \left({\mathsf{QALY}}_{\mathsf{TrueNegative}}-{\mathsf{QALY}}_{\mathsf{FalsePositive}}\right)-\left({\mathsf{Cost}}_{\mathsf{TrueNegative}}-{\mathsf{Cost}}_{\mathsf{FalsePositive}}\right)\right]$ is the INMB of preventing a false-positive diagnosis, $\varDelta \mathsf{TestCos}\mathsf{t}$ is the difference in cost between the new and old diagnostic strategies, and $\varDelta \mathsf{TestQALY}$ is the difference in the short-term quality-of-life effects associated with the test strategy.

## Results

### Lifetime costs and benefits of each diagnostic outcome

The lifetime discounted costs and QALYs associated with each possible diagnosis are shown in Table 2. Accurate diagnosis of AD decreased lifetime discounted costs by $9954 and increased lifetime QALYs by 0.248. In non-AD patients, a false diagnosis of AD increased lifetime costs by $11,345 due to unnecessary treatment costs.

**Table 2:** Average per-patient lifetime discounted costs and quality-adjusted life-years, by diagnostic outcome and strategy

	Cost (U.S.$)	LYs	QALYs	Probability of each outcome
Lifetime discounted costs and benefits by diagnostic outcome				
AD				
True-positive	$298,632	6.781	2.916	8.9%
False-negative	$308,586	6.555	2.660	3.8%
Not AD				
False-positive	$294,732	9.157	5.048	5.2%
True-negative	$283,387	9.157	5.048	82.1%
Lifetime discounted costs and benefits by diagnostic strategy				
Do nothing	$286,587 (244,438 to 337,270)		4.745 (3.88 to 5.42)	
Biomarker analysis (BM)	$286,752 (244,044 to 337,163)		4.760 (3.89 to 5.44)	
Incremental (BM vs. do nothing)	$165 (−1865 to 1625)		0.015 (−0.011 to 0.051)	
Incremental cost-effectiveness ratio ($ per QALY gained)			$11,032^a^	

*Abbreviations: AD* Alzheimer’s disease, *LY* Life-year, *QALY* Quality-adjusted life-year

^a^The empiric distribution of incremental cost-effectiveness ratios (ICERs) over the 10,000 simulations identified a 40% probability that biomarker analysis (BM) will decrease costs and increase QALYs and a 7% probability that BM will increase costs and decrease QALYs, assuming an average AD prevalence of 12.7%. Therefore, the 95% CI over the ICER ranges from BM is cost-saving to BM is dominated. Empiric 95% CIs were estimated from 10,000 simulations in which all input parameters were varied simultaneously

### Effectiveness and cost-effectiveness of diagnostic alternatives

**Fig. 2:**
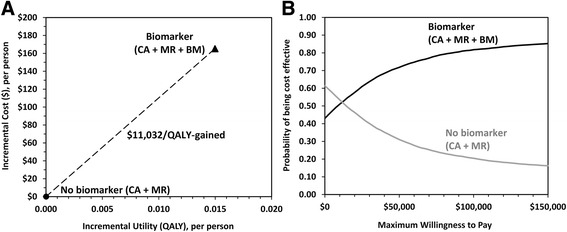
**a** Cost-effectiveness plane: incremental costs and incremental QALYs of CSF biomarker analysis. **b** Probability of each diagnostic strategy being the cost-effective alternative at various willingness-to-pay thresholds when all model input parameters are varied simultaneously. *AD* Alzheimer’s disease, *BM* Biomarker, *CA* Clinical assessment, *MR* Magnetic resonance, *QALY* Quality-adjusted life-year

At a 12.7% pretest probability of AD, biomarker analysis increased the average cost per patient by $165 (95% CI −$1865 to $1625) and increased the average QALYs per patient by 0.015 (95% CI −0.011 to 0.051). The relatively small gain in QALYs was due primarily to the short-term discomfort associated with the lumbar puncture procedure (−0.008 QALY), which was experienced by all patients. At this pretest probability of AD, the ICER of biomarker analysis was $11,032 per QALY gained (Fig. 2a). Probabilistic analysis identified extremely high uncertainty: a 40% probability that biomarker analysis will decrease costs and increase QALYs, and a 7% probability that it will do the opposite (increase costs and decreased QALYs). Overall, at an expected pretest prevalence of 12.7%, biomarkers were identified as cost-effective in 72% of simulations using a WTP threshold of $50,000 per QALY gained and 82% of simulations using a WTP threshold of $100,000 per QALY gained (Fig. 2b).

**Fig. 3:**
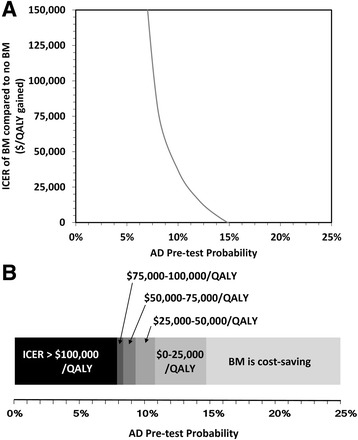
**a** Incremental cost effectiveness ratio (in U.S. dollars per QALY gained) of biomarker analysis for various pre-test probabilities of AD. **b** Incremental cost effectiveness ratio at various pre-test probabilities of AD. *AD* Alzheimer's disease, *BM* Biomarker, *ICER* Incremental cost-effectiveness ratio, *QALY* Quality-adjusted life-year

The results are highly influenced by the pretest prevalence of AD (Fig. 3a and b). The ICER rapidly increases as the pretest prevalence decreases (Fig. 3a); for pretest prevalence less than 9.1%, biomarker analysis costs more than $50,000 per QALY gained, and for pretest prevalence less than 7.5%, biomarker analysis costs more than $100,000 per QALY gained. For higher pretest prevalence, the ICER for biomarkers rapidly decreases, and for a pretest prevalence exceeding 15%, the probability that biomarkers are cost-effective is 74%, and deterministic analysis indicates biomarkers are cost-saving.

### Deterministic sensitivity analysis

At a pretest prevalence greater than 9%, deterministic sensitivity analysis indicated that biomarker analysis continued to be cost-effective within the ranges of uncertainty to disease progression rates, the rate of transition from living in the community to living in an LTCF, the cost of care in an LTCF, and the cost of biomarker testing. However, at a base case pretest prevalence of 12.7%, our findings were sensitive to patient age, rate of transition into an LTCF, the costs of long-term care, test performance, and treatment adherence (Additional file 1: Table S3). High rates of AD treatment adherence decrease the cost-effectiveness of biomarker analysis because they increase the costs associated with false-positive diagnoses. However, in a sensitivity analysis in which we considered that AD treatment may provide 50% of the benefit to patients with a false-positive diagnosis [59, 60], biomarker analysis costs more than $50,000 per QALY gained.

We relied heavily on the study of Bouwman et al. to estimate the sensitivity and specificity of biomarker analysis [13]. However, this study had relatively small sample size and used a gold standard of multidisciplinary team consensus rather than autopsy, the only true gold standard in AD diagnosis [61]. As such, we considered a wide range of sensitivities and specificities lower than in our base case (Additional file 1: Table S3). At moderately lower diagnostic accuracy (sensitivity 62%, specificity 92%), biomarker analysis remains the preferred alternative. At a low diagnostic accuracy (sensitivity 54%, specificity 89%), the ICER of biomarker analysis increases to $87,000 per QALY gained. Lowering the specificity further (sensitivity 54%, specificity 84%), the ICER of biomarker analysis exceeds $100,000 per QALY gained. Additionally, there is uncertainty about the proportion of patients who would receive a definitive non-AD diagnosis prior to biomarker analysis, which would increase the pretest prevalence of AD in the tested cohort. In this case of very low test accuracy, if AD prevalence in the tested cohort is 15%, the ICER is $87,600 per QALY gained.

**Fig. 4:**
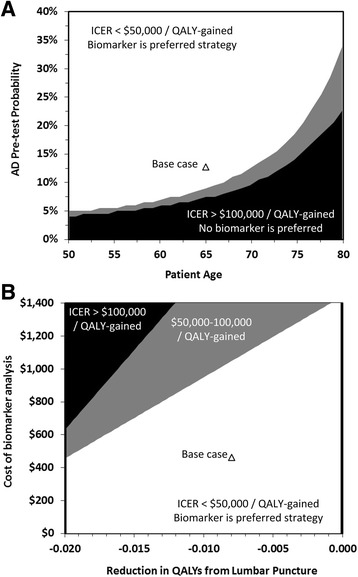
Two-way sensitivity analyses simultaneously varying (**a**) patient age and pre-test probability of AD and (**b**) utility detriment from lumbar puncture and the cost of biomarker analysis. *Triangle*: base case; *White* area: Cost saving at willingness-to-pay of 50,000 per QALY gained; *Grey* area: ICER of 50,000 100,000 per QALY gained; *Black* area: ICER> 100,000 per QALY gained. *AD* Alzheimer's disease, *ICER* Incremental cost-effectiveness ratio, *QALY* Quality-adjusted life-year

Two-way sensitivity analysis of prevalence and age revealed that, for younger patients, biomarker analysis is cost-effective at pretest probabilities of AD less than 8% at WTP of $50,000 per QALY gained (Fig. 4a). For older patients, such as those over the age of 75 years, biomarker analysis is cost-effective only in highly selected patient cohorts with pretest prevalence >27% and >20% at WTP of $50,000 or $100,000 per QALY gained, respectively (i.e., those with memory impairment). Two-way sensitivity analysis also identified that either increasing the cost of biomarker analysis by $1400 or increasing the utility decrement by 0.020 QALYs was sufficient for biomarker analysis to no longer be cost-effective (Fig. 4b).

Structural sensitivity analysis on the natural history of non-AD patients indicated that biomarker analysis is slightly more cost-effective if the conditions affecting patients without AD are more severe than we assumed in our base case. Biomarker analysis is less cost-effective if patients without AD but who are falsely diagnosed with AD receive a small benefit from acetylcholinesterase inhibitor treatment (Additional file 1: Table S3). Biomarker analysis is also less cost-effective if correction is made when disease progresses for patients with initially false-negative results (Additional file 1: Table S3).

### Challenge region

**Fig. 5:**
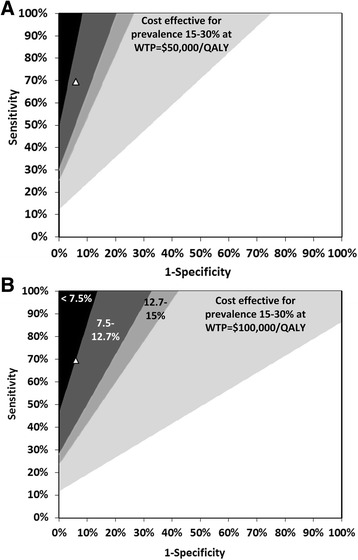
Challenge region. The challenge region identifies the test sensitivity-specificity combinations for a hypothetical new test, with a similar cost and short-term disutility as CSF biomarkers, that would be cost-effective compared to no test at four levels of pre-test prevalence: 7.5% (*black* area), 12.7% (*dark grey* area), 15% (*medium grey* area), and 30% (*light grey* area). The test accuracy of CSF biomarkers (*white triangle*) is also plotted for ease of comparing its test accuracy to the challenge region. (**a**) Willingness to pay threshold of 50,000 per QALY gained; (**b**) Willingness to pay threshold of 100,000 per QALY gained. *CSF* cerebrospinal fluid, *WTP* Willingness-to-pay, *QALY* Quality-adjusted life-year

When developing a diagnostic test, a trade-off exists between test sensitivity and specificity. In the case of AD, improved test sensitivity prevents delay in access to quality-of-life treatments caused by false-negative diagnoses (valued at $9954 per false-negative avoided), and improved test specificity prevents unnecessary treatment resulting from false-positive diagnoses (valued at $11,345 per false-positive avoided). The challenge region presented in Fig. 5 identifies the collection of all sensitivity and specificity pairs where a hypothetical test, with a cost and short-term disutility similar to those of CSF biomarkers, would be cost-effective compared with no test at four levels of pretest prevalence: 7.5%, 12.7%, 15%, and 30%.

## Discussion

For biomarker analysis to be cost-effective at a WTP of $50,000 per QALY gained, the pretest prevalence of AD in the tested cohort must be more than 1 in 11 patients. Overall prevalence of AD in the referral population varies substantially across referral centers, with specialized centers diagnosing AD in approximately one-fourth of referred patients [26, 61]. Evaluation of MTL atrophy by MRI will diagnose at least half of patients with AD. MRI may also identify a definitive diagnosis other than AD, which may preclude the need for continued evaluation in some patients. The optimal policy may therefore vary across clinics and may further depend on specific patient risk factors. In patients presenting to memory clinics with memory impairment without MTL atrophy, AD pretest prevalence may be greater than 14.5%; in these patients, biomarker analysis has the potential to be cost-saving. In addition to the benefits measured in the present study, timely diagnosis would also enable patients and their families to make informed decisions in planning future caregiving at a time when all parties achieve the greatest benefit and enable patients to have a greater role in making their own health care decisions before cognitive impairment interferes [62].

In practice, treatment uptake and adherence are low [63]. However, even with very low rates of treatment uptake and high rates of treatment discontinuation, biomarker analysis remains the preferred alternative (Additional file 1: Table S3). However, if patients without AD who receive a false-positive diagnosis of AD (and therefore initiate treatment at the same rates as patients with a true-positive diagnosis of AD) receive moderate benefits from donepezil and memantine for a disease with a similar progression to AD, biomarker analysis is no longer the cost-effective option at a WTP of $50,000 per QALY gained (Additional file 1: Table S3). This finding indicates that if patients with a false-positive diagnosis, for whom the cost of treatment will be incurred, receive a benefit from that treatment, the economic benefit derived from reducing the number of false-positives decreases. This finding does not indicate that donepezil or memantine treatment for patients without AD is necessarily cost-effective. The cost-effectiveness of cholinesterase inhibitor treatment in patients with non-AD disease has been demonstrated for Lewy body dementia [64], but acetylcholinesterase inhibitors have not shown clinical benefit for patients with MCI [65]. In general, the cost-effectiveness of a treatment depends on the natural history of the disease as well as the cost and efficacy of all treatment alternatives available to patients with that condition.

Studies in which researchers have estimated the diagnostic accuracy of clinical assessment, neuroimaging, and CSF biomarkers vary widely in their findings [66]. We used the study by Bouwman et al., who retrospectively applied each potential diagnostic strategy to 138 patients with AD and 223 memory clinic patients without AD [13]. Relying on a single study provided internally consistent estimates for the sensitivity and specificity of each test and the tests compared with each other, which may not have occurred had we collected test accuracy information from independent studies performed with different patient populations. At low pretest probabilities (<9%), the incremental cost of biomarker analysis was not robust to the uncertainty in test accuracy or many other input parameters. However, at higher pretest probabilities, the finding that biomarkers are cost-effective is robust to uncertainty in biomarker test accuracy (Fig. 5). This is relevant because specificity in particular may vary across referral centers, depending on the mix of patients composing the non-AD cohort. Greater confidence in the accuracy of diagnostic strategies can be established with larger sample size studies similar in design to that of Bouwman et al., in which multiple diagnostic criteria were applied to the same patients [13].

Our analysis has limitations, including a limited number of health states that do not fully represent the complex and multifaceted nature of AD and other neurological or psychiatric diseases represented in the non-AD population [18]. However, in addition to modeling cognitive functional decline, we included whether the patient was dwelling in the community or in an LTCF to incorporate elements of functional dependence, and we included disease severity-specific unpaid caregiving. Our inputs were derived from the medical literature. Specifically, transition rates for AD progression were based on an observational cohort not stratified by treatment status. In addition, several model parameters, including the accuracy of both diagnostic strategies, relied on studies with relatively small sample sizes and AD diagnosis based on clinical assessment, not on autopsy.

## Conclusions

Biomarker testing reduces the number of false-negative diagnoses and therefore connects patients to treatment earlier, improving their quality of life. Although the cost-effectiveness of biomarker analysis depends critically on the prevalence of AD in the tested population, it is cost-effective at a WTP of $50,000 per QALY gained in patient cohorts in which at least 1 (9%) in 11 patients has AD. In patients presenting to memory clinics with memory impairment without neuroimaging evidence of MTL atrophy, AD prevalence likely exceeds 15%. Biomarker analysis is potentially cost-saving and should be considered for adoption in high-prevalence centers.

## Additional file

Additional file 1: Cost-effectiveness of cerebrospinal biomarkers for Alzheimer’s diagnosis: supplemental Methods, Results, figures and tables as referenced in the text. (DOCX 91 kb)

## Abbreviations

AD: Alzheimer’s disease
Aβ: Amyloid-β
BM: Biomarker
CA: Clinical assessment
CPT: Current Procedural Terminology
CSF: Cerebrospinal fluid
CT: Computed tomography
HCPCS: Healthcare Common Procedure Coding System
ICER: Incremental cost-effectiveness ratio
INMB: Incremental net monetary benefit
LTCF: Long-term care facility
LY: Life-year
MCI: Mild cognitive impairment
MR: Magnetic resonance
MRI: Magnetic resonance imaging
MTL: Medial temporal lobe
NACC: National Alzheimer’s Coordinating Center
PET: Positron emission tomography
QALY: Quality-adjusted life-year
SN: Sensitivity
SP: Specificity
SPECT: Single-photon emission computed tomography
WTP: Willingness to pay
