# Supplementary material for: Cost-effectiveness of cerebrospinal biomarkers for the diagnosis of Alzheimer’s disease
Source: Alzheimers Res Ther. 2017 Mar 16;9:18. doi: 10.1186/s13195-017-0243-0 (PMC5356269; doi:10.1186/s13195-017-0243-0)
Supplement: Additional file 1: — Cost-effectiveness of cerebrospinal biomarkers for Alzheimer’s diagnosis: supplemental Methods, Results, figures and tables as referenced in the text. (DOCX 91 kb) [file 13195_2017_243_MOESM1_ESM.docx]

**Cost effectiveness of cerebrospinal biomarkers for Alzheimer’s diagnosis: Supplementary Material**

Lee SAW, Sposato LA, Hachinski V, Cipriano LE

1. **SUPPLEMENTAL METHODS**
   1. **Alzheimer’s disease transition probability calculations**

Spackman et al. estimated the probability of transitions between Alzheimer’s disease health states using the Uniform data set from the National Alzheimer’s Coordinating Center (NACC) in which some patients in each health state were receiving treatment [1]. Our model separately included age-specific mortality adjusted for disease severity, explicitly modeled the effectiveness of disease-modifying treatments, and explicitly modeled living in a long-term care facility (LTCF) accounting for different rates of treatment use among patients in LTCFs. We, therefore, sought to use the transition probabilities estimated by Spackman et al. to estimate treatment-stratified transition probabilities conditional on survival. We briefly explain the steps we took and the assumptions we made:

The following table is reproduced from Spackman et al. and presents the annual probability of transitions between states [1]:

|  | **Transition to** | | | |
| --- | --- | --- | --- | --- |
| **Transition from** | **Mild AD** | **Moderate AD** | **Severe AD** | **Dead** |
| **Mild AD** | 77.4% | 15.8% | 1.3% | 5.5% |
| **Moderate AD** | 7.0% | 50.1% | 21.4% | 21.5% |
| **Severe AD** | 0.2% | 2.7% | 49.1% | 48.0% |
| **Dead** | 0% | 0% | 0% | 100.0% |

First, we calculated the transition probabilities conditional on survival using the formula:

| *Probability of transition from state A to state B conditional on survival* | *=* | *Probability of transition from state A to state B* |
| --- | --- | --- |
|  |  | *Probability of survival from state A* |

For example, the probability of transitioning from Mild AD to Moderate AD (conditional on survival) is 0.158/(1-0.055) = 0.1672.

The following table presents the transition probabilities conditional on survival:

|  | **Transition to** | | |
| --- | --- | --- | --- |
| **Transition from** | **Mild AD** | **Moderate AD** | **Severe AD** |
| **Mild AD** | 81.90% | 16.72% | 1.38% |
| **Moderate AD** | 8.92% | 63.82% | 27.26% |
| **Severe AD** | 0.38% | 5.19% | 94.42% |

Spackman et al. also reported the proportion of the population receiving AD treatment stratified by disease severity [1]:

|  | **Receiving AD treatment** |
| --- | --- |
| **Mild AD** | 67.95% |
| **Moderate AD** | 79.85% |
| **Severe AD** | 66.51% |

Using the average annual transition probability between states, we calculated the annual transition rate using the formula:

*Annual Rate = – LN (1-Annual Transition Probability)*

The following table presents the annual transition rates between health states, conditional on survival, per 100,000 person years:

|  | **Transition to** | | |
| --- | --- | --- | --- |
| **Transition from** | **Mild AD** | **Moderate AD** | **Severe AD** |
| **Mild AD** |  | 18,296 | 1,385 |
| **Moderate AD** | 9,340 |  | 31,829 |
| **Severe AD** | 385 | 5,332 |  |

We do not calculate or present the annual rate of remaining in the same health state as we will calculate the transition probability for remaining as one minus the probabilities of transitioning to other states.

Consistent with a previous model-based analyses of AD, we assumed that AD treatment reduced the transition rate from mild to moderate AD with a hazard ratio of 0.50 and increased the transition rate from moderate to mild AD with a hazard ratio of 2.36 [2].

Using these assumptions for the influence of treatment on transition rates and the proportion of individuals receiving treatment, we calculated the annual transition rates stratified by whether the person was receiving AD treatment using the formula:

| *Average Rate* | = | *(Proportion not receiving AD treatment* × *Transition rate in AD patients not receiving AD treatment)* |
| --- | --- | --- |
|  |  | + |
|  |  | *(Proportion receiving AD treatment* × *Transition rate in AD patients not receiving AD treatment x Hazard ratio for transition rate in patients receiving treatment compared to those who are not receiving treatment)* |

We can rearrange this formula for the single unknown value, the transition rate in AD patients not receiving AD treatment:

| *Transition rate in AD patients not receiving AD treatment* | = | *Average Rate* |
| --- | --- | --- |
|  |  | *(Proportion not receiving AD treatment + (Proportion receiving AD treatment* × *Hazard ratio for transition rate in patients receiving treatment compared to those who are not receiving treatment)* |

An example calculation for the transition from Mild AD to Moderate AD is:

| *Transition rate in AD patients not receiving AD treatment* | = | *18296* | = | *27,710* |
| --- | --- | --- | --- | --- |
|  |  | *((1–67.95%) + (67.95%* × *0.5)* |  |  |

This resulted in calculating two transition rate matrices: one for patients who are not receiving treatment and one for patients who are receiving AD treatment.

Transition rates per 100,000 person years for patients not receiving treatment:

|  | **Transition to** | | |
| --- | --- | --- | --- |
| **Transition from** | **Mild AD** | **Moderate AD** | **Severe AD** |
| **Mild AD** |  | 27,710 | 1,385 |
| **Moderate AD** | 4,478 |  | 31,829 |
| **Severe AD** | 385 | 5,332 |  |

Transition rates per 100,000 person years for patients receiving treatment:

|  | **Transition to** | | |
| --- | --- | --- | --- |
| **Transition from** | **Mild AD** | **Moderate AD** | **Severe AD** |
| **Mild AD** |  | 13,855 | 1,385 |
| **Moderate AD** | 10,567 |  | 31,829 |
| **Severe AD** | 385 | 5,332 |  |

Finally, we converted these to monthly transition probabilities using the formula:

*Monthly transition probability = 1- EXP (-Annual rate/12 months per year)*

Monthly transition probabilities for patients not receiving treatment:

|  | **Transition to** | | |
| --- | --- | --- | --- |
| **Transition from** | **Mild AD** | **Moderate AD** | **Severe AD** |
| **Mild AD** | 97.60% | 2.28% | 0.12% |
| **Moderate AD** | 0.37% | 97.01% | 2.62% |
| **Severe AD** | 0.03% | 0.44% | 99.52% |

Monthly transition probabilities for patients receiving treatment:

|  | **Transition to** | | |
| --- | --- | --- | --- |
| **Transition from** | **Mild AD** | **Moderate AD** | **Severe AD** |
| **Mild AD** | 98.74% | 1.15% | 0.12% |
| **Moderate AD** | 0.88% | 96.51% | 2.62% |
| **Severe AD** | 0.03% | 0.44% | 99.52% |

We followed a similar set of steps to calculate treatment-stratified transition probabilities for the transition from residing in the community to residing in a LTCF.

Spackman et al. reported the average annual probability of transition from the community to a LTCF such as nursing homes as presented in the following table [1]:

|  | **Annual probability of transition to LTCF** |
| --- | --- |
| **Mild AD** | 0.012 |
| **Moderate AD** | 0.034 |
| **Severe AD** | 0.066 |

Beusterien et al. (2004) found that individuals not receiving acetylcholinesterase inhibitors (AChEI) for AD had a hazard ratio of 2.7 (95%CI 1.8-4.0) for transition to a nursing home compared to individuals on acetylcholinesterase inhibitor treatment using a retrospective analysis of a large US medical claims database including more than 5000 patients [3]. These findings are consistent with other studies, which have also found that acetylcholinesterase inhibitor treatment delays nursing home placement [4–6].

Following a similar procedure to the one we described above, we used the average annual transition probability for transition from community to LTCF, the proportion of individuals receiving treatment, and the influence of treatment on the transition to calculated the annual rate of transition to a LTCF stratified by treatment status using the formula:

| *Average Rate* | = | *(Proportion receiving AD treatment x Transition rate from the community to LTCF in AD patients receiving AD treatment)* |
| --- | --- | --- |
|  |  | + |
|  |  | *(Proportion not receiving AD treatment x Transition rate from the community to LTCF in AD patients receiving AD treatment x Hazard ratio for the transition rate to LTCF for patients not receiving treatment compared to those who are receiving treatment)* |

Rearranging this formula for the single unknown value (the transition rate to LTCF in AD patients receiving AD treatment) results in:

| *Transition rate from the community to LTCF in AD patients receiving AD treatment* | = | *Average Rate* |
| --- | --- | --- |
|  |  | *(Proportion receiving AD treatment + Proportion not receiving AD treatment x Hazard ratio for transition to LTCF for patients not receiving treatment compared to those who are receiving treatment)* |

Rates (per 100,000 person years) for transition from residing in the community to residing in a LTCF for the overall population, patients not receiving treatment, and those who are receiving treatment stratified by disease severity:

|  | **Annual rates (per 100,000 person years)** | | |
| --- | --- | --- | --- |
|  | **Average annual rate of transition to LTCF** | **Annual rate of transition to LTCF in patients not on AD treatment** | **Annual rate of transition to LTCF in patients on AD treatment** |
| **Mild AD** | 1,207 | 2,110 | 781 |
| **Moderate AD** | 3,459 | 6,957 | 2,577 |
| **Severe AD** | 6,828 | 11,747 | 4,351 |

We then converted the transition rates into monthly transition probabilities, shown in the following table:

|  | **Monthly probability of transition from residing in the community to residing in a LTCF** | |
| --- | --- | --- |
|  | **Not on AD treatment** | **On AD treatment** |
| **Mild AD** | 0.18% | 0.07% |
| **Moderate AD** | 0.58% | 0.21% |
| **Severe AD** | 0.97% | 0.36% |

- 1. **Cost of AD stratified by disease severity and location (community dwelling or living in a long-term care facility)**

Inpatient costs: Inpatient costs were calculated using common diagnoses for patient visits grouped by Diagnosis-Related Group (DRG) found in the Health Care Cost and Utilization Project database (HCUP). The Medicare cost by DRG was multiplied by the percent of hospitalized AD patients per specific DRG to get the average annual Inpatient cost per capita.

Outpatient costs: Outpatient costs were estimated based on a study from Zhao *et al.* (2004) that compared annual health care costs of AD patients to demographically matched patients in community and hospital settings [7].

Emergency care costs: Emergency costs by Alzheimer’s severity were calculated from the estimated emergency cost per visit and the proportion of patients within the state that required an emergency visit. Emergency costs were estimated using a study from Fillenbaum *et al* [8]*.* In this study, the authors calculated probability of at least one visit, frequency of visits, length of stay, and Medicare costs of hospitalization for 428 AD patients from the CERAD database. The proportion of patients within each disease state incurring emergency costs were estimated using a study by Gustavsson *et al.* that conducted interviews with 1222 patients from multinational AD patient cohort and stratified the results based on location of care (community or LTCF) and disease severity [9].

Unpaid caregiving: Informal care costs were calculated based on the average home care worker wage in the United States and multiplied by the estimated home care hours specific to disease severity. Although families continue to provide informal caregiving after patients move into long-term care facilities, additional informal costs were not counted for these patients. We assumed that the full cost of caregiving is accounted for in the cost of a LTCF even if all of the care is not provided by the facility. For example, family members may provide assistance with feeding a disabled patient but this task would be performed by a paid caregiver if the family member were not available and so the cost of doing so is already incorporated into the cost of the long-term care facility.

A complete breakdown of the severity-specific costs of AD care is presented in **Table S1.**

**Table S1 – Annual AD-severity specific cost breakdown (US$)**

|  | **Community dwelling** | | | **Long term care facility dwelling** | | | |
| --- | --- | --- | --- | --- | --- | --- | --- |
| **Cost category** | **Mild** | **Moderate** | **Severe** | **Mild** | **Moderate** | **Severe** | **Sources** |
| Inpatient | 6,995 | 6,995 | 6,995 | 6,995 | 6,995 | 6,995 | [8,10,11] |
| Outpatient | 2,813 | 2,813 | 2,813 | 2,813 | 2,813 | 2,813 | [7] |
| *Emergency care* |  |  |  |  |  |  |  |
| Cost per visit | 500 | 500 | 500 | 500 | 500 | 500 | [8] |
| Proportion of population that use emergency care | 2.9% | 2.5% | 2.1% | 12.7% | 12.7% | 7.8% | [9] |
| Average cost of emergency care | 15 | 13 | 11 | 64 | 64 | 39.00 |  |
| *Unpaid caregiving* |  |  |  |  |  |  |  |
| Caregiver hourly wage | 21 | 21 | 21 | N/A | N/A | N/A | [12] |
| Home care hours (hrs/week) | 13.1 | 22.0 | 46.1 | N/A | N/A | N/A | [13] |
| Average annual cost of unpaid caregiving | 14,305 | 24,024 | 50,341 | N/A | N/A | N/A |  |
| Total: | 24,128 | 33,845 | 60,160 | 9,872 | 9,872 | 9,847 |  |

- 1. **Probability distributions for probabilistic sensitivity analysis**

Probability distributions were assigned to all model inputs for probabilistic sensitivity analysis (**Table S2**). In most cases, probabilities and utility weights are modeled using Beta distributions, rates and costs are modeled using Normal distributions, and when the uncertainty around a parameter was highly skewed (such as for a hazard ratio), it was modeled using a Gamma distribution. A multivariate Normal distribution was used to simulate the joint distribution of biomarker test performance (described in detail below).

Distribution parameters were selected to maintain the same average value as the base case and to have approximately 95% confidence intervals based on the range used in the deterministic sensitivity analysis.

In some cases, we imposed additional rules to ensure logical rank ordering of randomly drawn values. For example, the quality-of-life utility weight with severe AD must be less than the utility weight with moderate AD which also must be less than the utility weight with mild AD.

Uncertainty in the proportion of patients without AD who will be correctly identified as having an alternative diagnosis (and not having AD) after MR causes the uncertainty in the pre-test prevalence of AD in our patient cohort to be right skewed. Therefore, we modeled it using a gamma distribution:


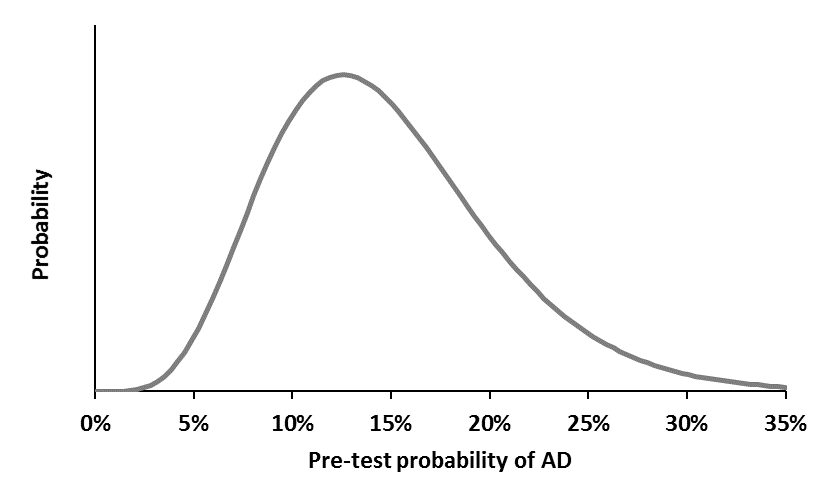


***Developing a joint probability distribution for test accuracy***

We used a multivariate Normal distribution in order to maintain a negative correlation between the sensitivity and specificity of the biomarker in probabilistic analysis. We used simulation to replicate the average values and the 95% confidence intervals reported in Bouwman et al. [14] using simple rules. Using the covariance matrix estimated by our simulation, we then assumed a multivariate Normal distribution using the base case values as the mean for each input:

Covariance matrix:

|  | sensitivity | specificity |
| --- | --- | --- |
| sensitivity | 0.00661 | (0.00026) |
| specificity | (0.00026) | 0.00064 |

The correlation coefficient between the sensitivity and specificity of the biomarker test was therefore –0.13.

**Table S2 – Base case inputs, ranges for sensitivity analysis, and sources.**

| **Parameter** | **Base case** | **Distribution for PSA*** |
| --- | --- | --- |
| **Patient population** |  |  |
| Start age (years) | 65 | *Not varied in PSA* |
| Proportion of referred population with Alzheimer’s Disease (AD) (%) | 12.7 | *See Supplementary Methods Section 1.3* |
| *Initial AD severity distribution (%)* |  |  |
| Mild | 70 | Beta(70, 30)** |
| Moderate | 28 | Beta(28, 78)** |
| Severe | 2 | Beta(2, 98)** |
|  |  |  |
| **Diagnosis** |  |  |
| Diagnostic Test Accuracy |  |  |
| Biomarker analysis (BM) |  |  |
| Sensitivity | 0.698 | *See Supplementary Methods Section 1.3* |
| Specificity | 0.941 | *See Supplementary Methods Section 1.3* |
| Costs |  |  |
| BM | 463 | Normal(356, 100) |
| Reduction in quality of life from test encounter and invasiveness |  |  |
| BM | -0.008 | -Beta(1, 125) |
|  |  |  |
| **AD natural history model** |  |  |
| Mortality |  |  |
| *Hazard ratios for AD-specific mortality* |  |  |
| Mild | 2.92 | Normal(2.92, 0.29) |
| Moderate | 3.85 | Normal(3.85, 0.46) |
| Severe | 9.52 | Normal(9.52, 1.5) |
| Disease progression without AD treatment (annual rate per 100,000) |  |  |
| From Mild |  |  |
| To Moderate | 27,710 | Normal(27710, 1386) |
| To Severe | 1,385 | Normal(1385, 69) |
| From Moderate |  |  |
| To Mild | 4,478 | Normal(4478, 224) |
| To Severe | 31,829 | Normal(31829, 1591) |
| From Severe |  |  |
| To Mild | 385 | Normal(385, 19) |
| To Moderate | 5,332 | Normal(5332, 267) |
| Transition to Long Term Care Facility Care (annual rate per 100,000) |  |  |
| From Mild | 2,110 | Normal(2110, 805) |
| From Moderate | 6,957 | Normal(6957, 2728) |
| From Severe | 11,747 | Normal(11747, 4624) |
|  |  |  |
| **AD Treatment** |  |  |
| Treatment uptake and adherence |  |  |
| *Treatment initiation* |  |  |
| Donepezil, at diagnosis | 0.45 | Beta(16.4,20) |
| Memantine, at transition to severe AD (with diagnosis) | 0.36 | 80% of donepezil |
| *Treatment discontinuation (annual rate per 100,000)* |  |  |
| Donepezil, community-dwelling | 28,768 | Normal(22047, 9116) |
| Donepezil, facility-dwelling | 62,362 | Normal(62362, 7992) |
| Memantine | 28,768 | Normal(22047, 5640) |
| *Treatment re-initiation after quitting (annual rate per 100,000)* |  |  |
| Donepezil | 33,142 | Normal(33142, 5018) |
| Memantine | 22,314 | Normal(22314, 2240) |
| Treatment effectiveness |  |  |
| *Donepezil (hazard ratios)* |  |  |
| Transition from mild to moderate | 0.5 | Gamma(5, 0.1) |
| Transition from moderate to mild | 2.36 | Gamma(4.5, 0.5) |
| Transition from community to long-term care facility | 0.37 | 1/Gamma(15, 0.18) |
| *Memantine* |  |  |
| Incremental utility (annualized) | 0.051 | Beta(5.1, 94.9) |
| Hazard ratio, transition from community to long-term care facility | 0.37 | 1/Gamma(15, 0.18) |
|  |  |  |
| **Costs** (2013 US$) |  |  |
| *Age Specific Baseline Costs* |  | Base case value × Normal(1, 0.1) |
| *Annual incremental costs by disease-severity (including costs of informal caregiving)* |  |  |
| Community dwelling |  |  |
| Patients without AD | 24,128 | Normal(24127, 1428) |
| Mild AD | 24,128 | Normal(24127, 1428) |
| Moderate AD | 33,845 | Normal(33845, 2328) |
| Severe AD | 60,160 | Normal(60160, 4813) |
| Long-term care facility dwelling |  |  |
| Facility Cost | 83,950 | Normal(83950, 5525) |
| Patients without AD | 9,872 |  |
| Mild AD | 9,872 | Normal(9872, 426) |
| Moderate AD | 9,872 | Normal(9872, 426) |
| Severe AD | 9,847 | Normal(9842, 426) |
| *Medication (annual)* |  |  |
| Donepezil, 10 mg/day | 2,473 | Normal(2473, 907) |
| Memantine, 10 mg/day | 3,192 | Normal(3240, 1380) |
| *Age-specific annual health care costs in the year of death* |  |  |
| <90 years | 35,158 | Normal(35,158, 5000) |
| > 90 years | 25,455 | Normal(25455, 3000) |
|  |  |  |
| **Utilities** |  |  |
| *Age-specific weights* |  |  |
| 60-64 years | 0.83 | Beta(415, 86) |
| 65-69 years | 0.82 | MIN(Utility for age 60-64, Beta(412, 89)) |
| 70-74 years | 0.81 | MIN(Utility for age 65-69, Beta(406, 95)) |
| 75-79 years | 0.79 | MIN(Utility for age 70-74, Beta(395, 105) |
| >79 years | 0.74 | MIN(Utility for age 75-79, Beta(316, 84) |
| *Health-state specific weights* |  |  |
| Community dwelling |  |  |
| Patients without AD | 0.68 | Beta(34, 16) |
| Mild AD | 0.68 | Beta(34, 16) |
| Moderate AD | 0.54 | MIN(Utility for Mild AD, Beta(27, 23)) |
| Severe AD | 0.37 | MIN(Utility for Moderate AD, Beta(18.5, 31.5)) |
| Long-term care facility dwelling |  |  |
| Patients without AD | 0.71 | Beta(35.5, 14.5) |
| Mild AD | 0.71 | Beta(35.5, 14.5) |
| Moderate AD | 0.48 | MIN(Utility for Mild AD in a LTCF, Beta(24, 26)) |
| Severe AD | 0.31 | MIN(Utility for Moderate AD in a LTCF, Beta(15.5, 34.5)) |

* Beta distributions are parameterized Beta(a, b) where the mean is calculated a/(a+b). Normal distributions are parameterized Normal(mean, standard deviation). Gamma distributions are parameterized Gamma(shape, scale) where the mean is calculated as shape multiplied by scale. MIN( x, y ) indicates that the minimum of two values was taken. For example, for the age-specific utility weight for age 65-69 is MIN(Utility for age 60-64, Beta(412, 89)) which ensures that the age-specific utility for age 65-69 cannot be greater than the age specific utility weight for age 60-64.

** Re-scaled to ensure the sum of the three probabilities is one.

1. **SUPPLEMENTAL RESULTS**

**Table S3 – Deterministic Sensitivity Analysis. The incremental cost and incremental QALYs of CSF Biomarker analysis compared to no biomarker and the preferred alternative at willingness-to-pay thresholds of $50,000 per QALY-gained and $100,000 per QALY-gained. Negative incremental values indicate that no biomarker analysis costs more and/or provided more QALYs than the biomarker analysis.***

| **Scenario** | **BM compared to No BM** | | | **Preferred alternative at**  **willingness to pay threshold** | |  |
| --- | --- | --- | --- | --- | --- | --- |
|  | **Incremental Costs** | **Incremental QALYs** | **$50,000**  **/QALY-gained** | | **$100,000**  **/QALY-gained** | |
| **Base Case** | **165** | **0.015** | **BM** | | **BM** | |
| Age |  |  |  | |  | |
| Younger, Age 60 | -269 | 0.020 | BM | | BM | |
| Older, Age 75 | 653 | 0.005 | Do nothing | | Do nothing | |
| Older, Age 80 | 730 | 0.000 | Do nothing | | Do nothing | |
| AD Severity distribution at diagnosis |  |  |  | |  | |
| Shifted to less severe  (Mild = .78; Moderate = .21; Severe = .01) | 144 | 0.016 | BM | | BM | |
| Shifted to more severe diseases [4]  (Mild = .61; Moderate = .34; Severe = .04) | 191 | 0.014 | BM | | BM | |
| Biomarker test accuracy |  |  |  | |  | |
| Very low accuracy and moderately high prevalence: (SN=54%, SP=84%, Prevalence=15%) | 1,161 | 0.013 | Do nothing | | BM | |
| Very low: (SN=54%, SP=84%) | 1,326 | 0.010 | Do nothing | | Do nothing | |
| Low: (SN=54%, SP=89%) | 870 | 0.010 | Do nothing | | BM | |
| Moderately low: (SN=62%, SP=92%) | 518 | 0.012 | BM | | BM | |
| Moderately high: (SN=78%, SP=96%) | -130 | 0.018 | BM | | BM | |
| High: (SN=86%, SP=98%) | -426 | 0.020 | BM | | BM | |
| BM test cost |  |  |  | |  | |
| Low cost: ($250) | -48 | 0.015 | BM | | BM | |
| High cost: ($600) | 302 | 0.015 | BM | | BM | |
| BM quality of life decrement |  |  |  | |  | |
| Low: (no decrement) | 165 | 0.023 | BM | | BM | |
| High: (-0.02) | 165 | 0.003 | BM | | BM | |
| Treatment |  |  |  | |  | |
| Treatment decreases moderate to severe transition | 299 | 0.016 | BM | | BM | |
| All patients with AD diagnosis initiate treatment | -141 | 0.023 | BM | | BM | |
| High treatment uptake (56%) | 104 | 0.017 | BM | | BM | |
| Low treatment uptake (27%) | 265 | 0.012 | BM | | BM | |
| Low treatment uptake and poor adherence | 281 | 0.011 | BM | | BM | |
| Best case scenario: high uptake, high adherence, low cost and high effectiveness (utility benefit and impact on transition rates) | -852 | 0.059 | BM | | BM | |
| Worst case scenario: low uptake, poor adherence, high cost and low effectiveness | 1356 | -0.007 | Do nothing | | Do nothing | |
| Discontinuation Rate^**^ |  |  |  | |  | |
| High (% per year) | 191 | 0.013 | BM | | BM | |
| Low (% per year) | 82 | 0.022 | BM | | BM | |
| No Discontinuation | 206 | 0.029 | BM | | BM | |
| Proportion of false negative diagnoses corrected when patients’ disease progresses | | | | | | |
| 50% | 314 | 0.011 | BM | | BM | |
| 75% | 386 | 0.009 | BM | | BM | |
| 100% | 456 | 0.007 | Do nothing | | BM | |
| Mortality |  |  |  | |  | |
| Low for all severity levels | -270 | 0.017 | BM | | BM | |
| High for all severity levels | 509 | 0.013 | BM | | BM | |
| AD disease progression |  |  |  | |  | |
| No backwards transitions | 379 | 0.011 | BM | | BM | |
| 10% slower disease progression | 140 | 0.015 | BM | | BM | |
| 10% faster disease progression | 186 | 0.015 | BM | | BM | |
| Severity specific quality of life |  |  |  | |  | |
| Low for all AD states | 165 | 0.009 | BM | | BM | |
| High for all AD states | 165 | 0.014 | BM | | BM | |
| Caregiving and long term care facility (LTCF) |  |  |  | |  | |
| High caregiving costs | 325 | 0.015 | BM | | BM | |
| High LTCF accommodation costs | -208 | 0.015 | BM | | BM | |
| Lower transition rate to LTCF for all severity levels | 562 | 0.015 | BM | | BM | |
| Higher transition rate to LTCF for all severity levels | -381 | 0.015 | BM | | BM | |
| High accommodation and caregiving costs and high transition rate to LTCF | 930 | 0.014 | Do nothing | | BM | |
| Natural History of non-AD disease |  |  |  | |  | |
| Stable moderate disease | -3 | 0.009 | BM | | BM | |
| AD-like progression | 499 | -0.007 | Do nothing | | Do nothing | |
| AD-like progression and AD treatment is partially effective in non-AD patients*** | 588 | -0.090 | Do nothing | | Do nothing | |

*Sensitivity analysis was performed at a prevalence rate of 12.7%, except for the scenarios testing high and low AD prevalence values.

**High discontinuation % per year: Community-donepezil 30%, LTCF-donepezil 50%, Community and LTCF memantine 36%; Low discontinuation % per year: Community-donepezil 10%, LTCF-donepezil 40%, Community and LTCF memantine 12%.

***Falsely diagnosed non-AD patients (False positive result) undergo 75% slower AD-like progression, Donepezil is 75% as effective, Memantine is 75% as effective and 50% of the base case value for restarting treatment.

1. **REFERENCES**

1. Spackman D, Kadiyala S, Neumann PJ, Veenstra D, Sullivan S. Measuring Alzheimer Disease Progression with Transition Probabilities: Estimates from NACC-UDS. Curr. Alzheimer Res. 2012;9:1050–8.

2. Neumann PJ, Hermann RC, Kuntz KM, Araki SS, Duff SB, Leon J, et al. Cost-effectiveness of donepezil in the treatment of mild or moderate Alzheimer’s disease. Neurology. 1999;52:1138–45.

3. Beusterien KM, Thomas SK, Gause D, Kimel M, Arcona S, Mirski D. Impact of Rivastigmine Use on the Risk of Nursing Home Placement in a US Sample. CNS Drugs. 2004;18:1143–8.

4. Feldman HH, Pirttila T, Dartigues JF, Everitt B, Van Baelen B, Schwalen S, et al. Treatment with galantamine and time to nursing home placement in Alzheimer’s disease patients with and without cerebrovascular disease. Int. J. Geriatr. Psychiatry. 2009;24:479–88.

5. Geldmacher DS, Provenzano G, McRae T, Mastey V, Ieni JR. Donepezil is associated with delayed nursing home placement in patients with Alzheimer’s disease. J. Am. Geriatr. Soc. United States; 2003;51:937–44.

6. Wattmo C, Wallin AK, Londos E, Minthon L. Risk factors for nursing home placement in Alzheimer’s disease: a longitudinal study of cognition, ADL, service utilization, and cholinesterase inhibitor treatment. Gerontologist. United States; 2011;51:17–27.

7. Zhao Y, Kuo T-C, Weir S, Kramer MS, Ash AS. Healthcare costs and utilization for Medicare beneficiaries with Alzheimer’s. BMC Health Serv. Res. 2008;8:108.

8. Fillenbaum G, Heyman A, Peterson B, Pieper C, Weiman AL. Frequency and duration of hospitalization of patients with AD based on Medicare data: CERAD XX. Neurology. 2000;54:740–3.

9. Gustavsson A, Brinck P, Bergvall N, Kolasa K, Wimo A, Winblad B, et al. Predictors of costs of care in Alzheimer’s disease: a multinational sample of 1222 patients. Alzheimers. Dement. 2011;7:318–27.

10. Centers for Medicare & Medicaid Services. Medicare Fee-for-Service Payment Schedule (2009). Available at: https://www.cms.gov/Medicare/Medicare.html.

11. Agency for Healthcare Research and Quality. HCUPnet, Healthcare Cost and Utilization Project. 2012.

12. MetLife. Market Survey of Long-Term Care Costs. 2012.

13. Langa KM, Chernew ME, Kabeto MU, Herzog a R, Ofstedal MB, Willis RJ, et al. National estimates of the quantity and cost of informal caregiving for the elderly with dementia. J. Gen. Intern. Med. 2001;16:770–8.

14. Bouwman FH, Verwey NA, Klein M, Kok A, Blankenstein MA, Sluimer JD, et al. New Research Criteria for the Diagnosis of Alzheimer’s Disease Applied in a Memory Clinic Population. Dement. Geriatr. Cogn. Disord. 2010;30:1–7.
